# Recent Advances in the Diagnosis and Treatment of Clostridium Difficile Infection

**DOI:** 10.12688/f1000research.7109.1

**Published:** 2016-01-29

**Authors:** Meera B. Avila, Nathaniel P. Avila, Andrew W. Dupont

**Affiliations:** 1 Department of Gastroenterology, Hepatology and Nutrition, University of Texas Medical School at Houston, Houston, TX, 77030, USA

**Keywords:** Clostridium Difficile, CDI, antibiotics, microbiota

## Abstract

*Clostridium difficile* infection (CDI) has become the most frequently reported health care-associated infection in the United States [1]. As the incidence of CDI rises, so too does the burden it produces on health care and society. In an attempt to decrease the burden of CDI and provide the best outcomes for patients affected by CDI, there have been many recent advancements in the understanding, diagnosis, and management of CDI. In this article, we review the current recommendations regarding CDI testing and treatment strategies.

## Introduction

*Clostridium difficile* is an opportunistic organism that causes infection in patients with an alteration in intestinal microbiota. Microbiota is the community of organisms that inhabits a particular region of the body, and the intestine is composed of 300–500 species of bacteria. Alteration in intestinal microbiota predisposes patients to becoming infected with the spores from *C. difficile* via fecal-oral transmission ^2^. Once a patient has *C. difficile* infection (CDI), outcomes can range from asymptomatic colonization to severe diarrhea. Fulminant or severe complicated CDI is characterized by inflammatory lesions and the formation of pseudomembranes in the colon, which can lead to toxic megacolon, bowel perforation, sepsis, shock, and death ^2^. In addition, CDI has become nefarious for more severe disease associated with frequent recurrences despite appropriate and adequate treatment ^3^, in part due to a virulent strain of CD termed NAP1/B1/027 ^4^. The consequences of CDI affect the patient and society alike, as more than 300,000 hospitalizations involve CDI each year. The mean cost of each hospitalization ranges from $8911 to $30,049 per patient, at a yearly cost estimated at $1.0 to $4.9 billion to the US health care system ^5, 6^. While a large portion of this cost is related to a true increase in CDI incidence, some of the cost burden can be attributed to the over-diagnosis of CDI after the introduction of molecular tests ^7– 10^. As health care costs rise, so does the importance of continued research in the detection and treatment of CDI.

### Update in diagnosis of CDI

In the molecular era, how to best diagnose CDI in a cost-effective manner has become an area of much debate. In order to efficiently and effectively treat CDI, the diagnosis should be made rapidly based on clinical and laboratory evidence of the infection. Testing for CDI should only occur if patients have clinical risk factors for the disease along with signs and symptoms, most commonly diarrhea ^11^. The most common risk factors include patients who are currently receiving antibiotics or who have received antibiotics in the past 8 weeks ^12^. There are compelling data that almost all antibiotics can increase the risk of CDI, but third-generation cephalosporins, clindamycin, amoxicillin, and fluoroquinolones have been the most frequently reported ^12– 14^. In addition, patients are at greater risk if their age is greater than 65, if they are hospitalized or were recently hospitalized, or if they live in long-term care facilities ^4^.

Laboratory testing for CDI should be performed only on symptomatic patients and only on diarrheal stool ^15– 17^. Additionally, testing patients with CDI for “cure or clearance” or for “colonization” after treatment is not appropriate and not recommended ^16^. Treated patients often shed spores for several weeks to months despite being asymptomatic, and further testing can lead to inappropriate courses of treatment ^15, 17^. There is general consensus that radiologic diagnosis of CDI is of little value ^18^; however, imaging should be done in cases of suspected toxic megacolon. Endoscopic diagnosis should be reserved for cases when a diagnosis is emergently needed, if there is delay in implementing CDI testing, if laboratory tests are negative and CDI is strongly suspected, or in cases of ileus when stool is unavailable ^19^.

Laboratory testing for CDI is an exciting and rapidly changing field; however, it remains an area of confusion, largely because there is no generally accepted gold standard or single best test ^20^. In general, the clinical usefulness of a CDI diagnostic test is judged on its sensitivity, specificity, turnaround time (TAT), cost, and availability ^21^. Currently, the five accepted tests are enzyme immunoassay (EIA) for toxin A/B, glutamate dehydrogenase (GDH), nucleic acid amplification tests (NAATs), toxigenic culture (TC), and cytotoxin neutralization (CTN) test. These tests vary widely in terms of clinical usefulness ( Table 1) ^21^.

**Table 1.** Properties of tests available for *C. difficile* infection detection.

		Sensitivity	Turnaround Time (TAT)	Cost	Availability
Detects gene responsible for production of Toxin	NAATs ^1~^	***	Hours ^6^	****	***
Detects toxin in stool	CTN ^2+^	***	Days	***	* ^7^
	EIA ^3^ toxin A/B	**	Hours	*	****
Detects common antigen on *C. difficile*	GDH ^4^	****	Hours	*	****
Relies on culture of *C. difficile*	TC ^5^	*****	***	* ^7^	Days

1. NAAT: nucleic acid amplification test; 2. CTN: cytotoxin neutralization test; 3. EIA: solid-phase enzyme immunoassay; 4. glutamate dehydrogenase; 5. toxigenic culture; 6. TAT are variable and dependent on type of NAAT; 7. only available in specialty research laboratories; * indicates magnitude of characteristic, i.e. *** has a greater cost than **

Toxins A and B are the most important virulence determinants of disease and the majority of diagnostic tests target these toxins ^22^. These toxins are responsible for symptoms of infection and are present in the stool of infected patients with diarrhea. The first test for detection, the CTN test, was developed in the 1970s ^23^. CTN was novel in that it detected *C. difficile* toxins on cell culture medium. Unfortunately, CTN requires significant expertise, is time consuming, has very slow TAT, and is not widely available ^21, 24^.

Subsequently, TC on selective medium was developed for the detection of *C. difficile* ^23^. Although considered the gold standard for its time due to its very high sensitivity, it lacks specificity. Data now show a high rate of false positives in asymptomatic carriers and in certain patient populations, such as infants and patients recently exposed to antibiotics ^2^. In addition, it has a very slow TAT and is not widely available, as testing requires an experienced laboratory ^21^.

In the early 1990s, detection of *C. difficile* toxins A and B through solid-phase EIAs was developed. EIAs have a rapid TAT, are widely available and inexpensive, and thus became the new standard for CDI detection in most laboratories until the early 2000s ^21^. Although initially reported to have a sensitivity of as high as 98%, subsequent studies showed toxin A and B EIA had a poorer sensitivity, between 45 and 60%, respectively, but a positive predictive value between 90 and 100%, respectively ^22^. Currently, the general consensus is that the EIA for toxin A and B is too insensitive and is no longer recommended as a stand-alone test ^11^.

In 2006, the GDH assay was marketed as a CDI detection test. GDH detects *C. difficile* cell-wall-associated antigen and has a reported sensitivity of 100%. To its strength, GDH has a negative predictive value approaching 100%, but with a positive predictive value of only 59% ^25^. It has a rapid TAT, is widely available and affordable, and has become an effective screening tool for CDI detection ^21^. GDH, however, detects all *C. difficile*, including nontoxigenic strains, subsequently lowering the specificity for the diagnosis of CDI ^26^.

Given the high specificity of toxin A/B EIAs and the sensitivity of GDH, several laboratories adopted a two-step algorithm for testing. Referred to as a multistep approach, CDI testing begins with common antigen GDH. If GDH is found to be positive, the toxin A/B assay is performed for the detection of direct toxin production ^27^. GDH and EIA have subsequently been combined and marketed as a single confirmatory test for CDI. The C. Diff QUIK CHEK Complete assay (TechLab, Blacksburg, VA) combines GDH testing and toxin testing using a toxin A/B EIA ^28^. This assay takes about 30 minutes to perform and has a built-in control. At least two publications demonstrate sensitivities of 100% for the GDH portion of the test ^24, 25^. The combination of the two tests together in a step-wise process is recognized as confirmation of CDI ^15, 16^. Unfortunately, testing can produce discordant results, which can be difficult to interpret, and thus confirmation requires further diagnostic testing ^20, 21^.

Further advancements in detection came in 2009 when NAATs for CDI became commercially available ^29^. The basis of NAATs is the detection of toxigenic *C. difficile* strains based on DNA extraction from the stool ^21^. In general, the target of most NAATs is the gene responsible for coding toxin B ( *tcdB* gene) ^29, 30^. At this time, there are nine US Food and Drug Administration (FDA)-approved *C. difficile* NAATs. Six are polymerase chain reaction (PCR)-based assays and three are isothermal assays. The assays have sensitivities ranging from 80 to 100%, specificities ranging from 87 to 99%, and all have rapid TATs. NAATs have quickly become popular, and in many laboratories they have become a stand-alone approach for the diagnosis of CDI ^21, 29^. NAATs have also been shown to lead to a more rapid diagnosis when compared to GDH and EIA ^31^. There are some data showing earlier detection has led to fewer CDI-related complications, such as intensive care unit admission, colectomy, and death ^6^. However, NAATs have been criticized for being overly sensitive, and their use as a stand-alone test has been controversially linked to elevated reported incidence rates of CDI ^7, 20, 29^. False positives can occur with NAATs, as they do not detect the presence of biologically active toxin in stool specimens and can detect only the genes responsible for potential toxin production. This has led many to believe that over-diagnosis of colonized *C. difficile* patients is occurring and that NAATs have increased antibiotic treatment for possible colonized states or limited infections ^10, 20, 32^.

The best standard laboratory test for the diagnosis of CDI has not yet been defined; however, recent clinical guidelines on this topic have been published by the Society for Healthcare Epidemiology of America (SHEA), the Infectious Diseases Society of America (IDSA) ^15^, the American College of Gastroenterology (ACG) ^16^, and the United Kingdom National Health Service ^26^. In the United States, the ACG recommends the use of the NAAT as the best test for CDI diagnosis, either as a stand-alone test or as part of a multistep testing algorithm. The ACG also states GDH testing can be used in a two- to three-step algorithm that includes subsequent toxin A/B EIA testing ^16^. The IDSA recommends a two-step method that uses GDH as initial screening followed by the CTN or TC as a confirmatory test. The IDSA recognizes the potential value of NAATs; however, it does not currently recommend these tests in the diagnosis of CDI, citing more data on utility is necessary ^16^. In the United Kingdom, guidelines recommend a combination of two tests, the first of which should be a NAAT or GDH followed by a toxin EIA test ^33^. All guidelines make a significant contribution to clinical decision making, but recent updates should also be considered when choosing testing and treatment.

### Update in treatment of CDI

There has recently been much research in the field of CDI treatment. New medications and novel therapy highlight the progression made. The first step in treating CDI is to stop the offending antibiotic when possible. Although it is difficult in the age of polypharmacy to accurately quantify the association between antibiotics and CDI, most studies have determined a link between prior exposure to antimicrobial agents and CDI ^12– 14^. Medical treatment of CDI varies based on a graded severity scale and whether it is the first occurrence or recurrence of the disease. Severity is usually defined by factors such as age, temperature, serum albumin, and white blood cell count. Guidelines recommend the use of metronidazole 500 mg orally three times per day for 10–14 days for initial mild to moderate disease, and vancomycin 125 mg orally four times per day for 10–14 days for initial severe disease ^15, 16, 34^. The evidence for these guidelines is supported by Zar *et al.*’s randomized, prospective, double-blind, placebo-controlled trial, showing vancomycin to be superior to metronidazole in curing severe cases of CDI (97% vs. 76% of patients respectively, p=0.02) ^35^. In patients with mild disease, cure rates were similar in the two treatment groups ^35^. Another study comparing treatment of CDI with vancomycin or metronidazole in patients risk-stratified by infection severity showed significantly less treatment-refractory disease after treatment with vancomycin in severe cases of CDI (32% refractory disease in pre-implementation phase vs. 15% in post-implementation phase, p=0.035) ^36^. Additionally, in another randomized control trial, vancomycin was found to be superior to metronidazole in terms of clinical success and cure rates in patients with severe CDI (88% vs. 77% in the vancomycin and metronidazole groups, respectively) ^37^.

There is a high risk of recurrence associated with CDI. Studies show that up to 25–30% of patients appropriately treated for CDI experience at least one additional episode ^15, 34^. Recurrence comprises both episodes of relapse with infection by the current strain and reinfection by a new strain, and it remains difficult to distinguish between the two infections ^37, 38^. Treatment of the first episode of recurrence is usually with the same antibiotic used to treat the initial episode; however, treatment should also be guided by CDI severity if there is a significant change. To help combat the increasing burden of recurrence, the FDA approved fidaxomicin (FDX) for the treatment of CDI in 2011. FDX is a non-absorbed macrolide antibiotic effective against Gram-positive anaerobes but with no effect against bacteroides, a prominent constituent of the intestinal flora. Unlike metronidazole and vancomycin, which both have activity against bacteroides, FDX is thought to have some intestinal microbiota-sparing effect ^34^. FDX has been shown to be superior in the prevention of recurrence of CDI ^3, 39^. A large randomized control trial comparing FDX to vancomycin demonstrated a lower rate of recurrence in the FDX group ^34, 39^. In another randomized double-blind trial comparing FDX to vancomycin, clinical response rates were similar in the treatment of a first recurrence of CDI; however, FDX was shown to be more likely to prevent a second recurrence ^34^. There are concerns about the cost of FDX, as it is nearly 10-times more expensive than current standard oral vancomycin ^40^. However, a recent study assessed the economic impact of treatment with FDX compared to oral vancomycin and showed an overall cost benefit in patients treated with FDX ^40^. Patients treated with FDX had lower rates of recurrence, lower rates of hospital readmission, and shorter hospital stays, resulting in an overall saving of $3047 per patient treated with FDX ^40^. Although this study has its limitations, it promotes further advancements in the future of CDI treatment with reduced rates of recurrence.

One existing concept for the treatment of CDI that is gaining popularity is bacteriotherapy with fecal microbiota transplantation (FMT). FMT has been shown to be an effective treatment for recurrent CDI ^29^. Stool from a healthy donor in the form of a liquid suspension has traditionally been transplanted into the patient’s gastrointestinal tract. This can be performed through a variety of routes including nasogastric tube, nasojejunal tube, upper endoscopy, colonoscopy, or enema, with similar success rates ^41^. The rationale for FMT is to restore a healthier intestinal microbiota in patients with recurrent CDI who have disrupted intestinal flora and decreased microbiota diversity from antibiotic therapy ^42^. FMT was previously considered a therapy of last resort for CDI; however, there has been significant research and interest in FMT, and it is becoming more widely practiced ^41– 43^. A case series of 12 patients with recurrent CDI treated with FMT demonstrated a 100% cure rate ^44^. Another case series of 18 patients demonstrated a 94% cure rate in the 16 surviving patients ^45^.

A randomized control trial in 2013 compared FMT with donor feces solution transmitted via nasoduodenal tube preceded by four doses of vancomycin and bowel lavage vs. standard vancomycin with and without bowel lavage ^46^. This study showed resolution of diarrhea in 81% of patients after the first FMT and in 94% of patients overall, as two patients were subsequently cured after second infusion of donor feces. Comparatively, only 31% of patients in the vancomycin alone group and 23% in the vancomycin with bowel lavage group had resolution of diarrhea ^46^. Adverse events included diarrhea (94%) immediately after donor-feces infusion, as well as cramping (31%), constipation (19%), and belching (19%). No persistent adverse events related to FMT were noted. The most recent and largest systematic review with meta-analysis in 2015 of FMT studies, involving 18 observational studies with 611 patients, showed a primary cure rate of 91.2% (95% confidence interval [CI] 86.7–94.8%). The overall recurrence rate of CDI was 5.5% (95% CI 2.2–10.3%). The early recurrence rate and late recurrence rate were 2.7% (95% CI 0.7–6.0%) and 1.7% (95% CI 0.4–4.2%), respectively. Most adverse events were expected, short-lived, self-limited, and manageable ^47^. These studies seem to show that FMT is a highly effective therapy for recurrent CDI.

Another advancement has been the use of probiotics to prevent the development of CDI. Since antibiotics disturb the natural intestinal flora, leading to susceptibility to infection from *C. difficile*, a treatment which prevents alteration of the natural intestinal microbiome is theorized to help prevent CDI ^48^. The use of lactobacillus has been shown to reduce diarrheal symptoms and reduce the risk of CDI in hospitalized patients on antibiotics ^49^. A large meta-analysis (Cochrane review) composed of 23 randomized controlled trials with 4213 patients showed a significant relative risk reduction in the incidence of *C. difficile-*associated diarrhea in patients treated with probiotics ^49^. In contrast, a large prospective randomized control trial composed of 3981 patients compared the incidence of antibiotic-associated diarrhea, including *C. difficile*-associated diarrhea, in patients receiving probiotics compared to a placebo group and found similar incidences of antibiotic-associated diarrhea in the probiotic and placebo groups ^50^. Overall, strong evidence to support the use of probiotic use in the treatment or prevention of CDI is lacking. However, given the overall low cost and lack of significant side effects with probiotics, they are often used to attempt prevention of CDI in patients prescribed antibiotics.

In summary, many recent efforts and advancements have been made in the diagnosis and treatment of CDI. Rapid and accurate detection of CDI has improved significantly, but possibly at the cost of over-diagnosis. There is still no uniform agreement regarding the best means of diagnosing CDI. Also, when discordant results occur with testing, this may lead to confusion regarding therapy. Future treatment of CDI seems promising, as recent advancements in newer antibiotic therapy and FMT have been shown to more effectively treat CDI, especially in terms of lowering rates of recurrence and also in the treatment of recurrent infection. With the rising burden of CDI, continued research in diagnostic testing and treatment is needed to combat this significant health care problem.

## Abbreviations

CDI: Clostridium difficile infection

CI: confidence interval

CTN: cytotoxin neutralization test

EIA: enzyme immunoassay

FMT: fecal microbiota transplantation

GDH: glutamate dehydrogenase

NAAT: nucleic acid amplification test

TAT: turn around time
